# Supplementary material for: The Singular Molecular Conformation of Humic Acids in Solution Influences Their Ability to Enhance Root Hydraulic Conductivity and Plant Growth
Source: Molecules. 2020 Dec 22;26(1):3. doi: 10.3390/molecules26010003 (PMC7792592; doi:10.3390/molecules26010003)
Supplement: Supplementary file 1 [file molecules-26-00003-s001.pdf]

Supplementary material

The singular molecular conformation of humic acids in solution influences their ability to enhance root hydraulic conductivity and plant growth.

Maite Olaetxea [1], Veronica Mora [2], Roberto Baigorri [3], Angel M Zamarreño [4] and Jose M García-Mina [5]

[1]Department of Environmental Biology, BIOMA Institut, Sciences School, University of Navarra; molaetxea@unav.es

[2]Plant Physiology and plant-microorganism interaction laboratory. Universidad Nacional de Río Cuarto. Córdoba. Argentina; moraveronica@yahoo.com.ar

[3] Department of Environmental Biology, BIOMA Institut, Sciences School, University of Navarra; roberto.baigorri@tervalies.com

[4] Department of Environmental Biology, BIOMA Institut, Sciences School, University of Navarra; angelmarizama@unav.es

[5] Department of Environmental Biology, BIOMA Institut, Sciences School, University of Navarra; jgmina@unav.es

Humic acid MW characterization:

<u>DOSY H1 NMR</u> was performed to calculate the MW distribution of HA. A Nuclear Magnetic Resonance Bruker AVANCE AV-500 (500 MHz) was utilized using a 1H-broadband inverse probe. Spectrum was acquired by 128 scans with 2 ms pulses, with 100-200 ms diffusion time. Diffusion coefficients were calculated by using MestReC 4.7.0.0.© 2005 software.

Diffusion coefficients were calculated as follow:

$$I = I_0 \exp\left[-D_f(\Delta - \delta/3 - \tau/2)(g\gamma\delta)^2\right]$$

where I is resonance spectrum intensity, I_0 is resonance spectrum intensity without gradient pulse, is diffusion time, g y are gradient pulse amplitude and time respectively, pulses delay, H gyromagnetic radius: 26571 and D_f diffusion coefficient. Gradient intensity follows a decay line and mono exponential non-linear curve fit was applied for each DOSY spectrum peak,

$$f(x) = B \exp(-x F)$$

where F is the diffusion coefficient.

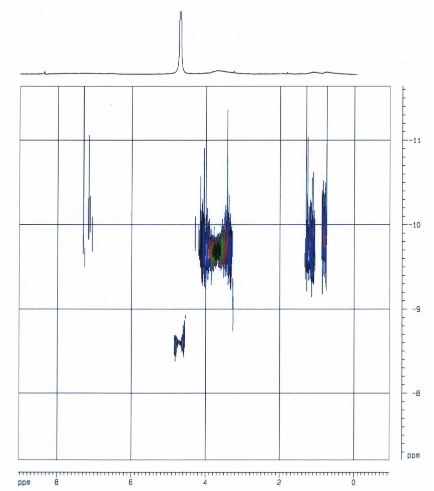

Figure S1. DOSY H1 NMR Spectrum from HA 100 mg L^{-1}

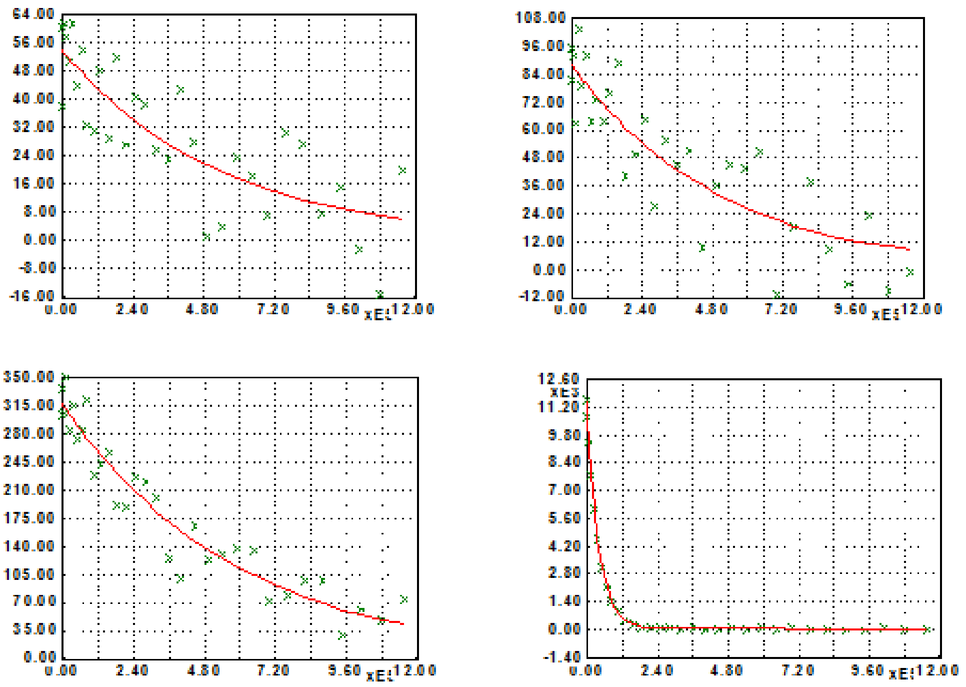

Dosy H1 NMR Peaks	D_f	MW
1.20 ppm	2,26 E-10	2163
2.14 ppm	1.14 E-10	9482
3.75 ppm	7.50 E-11	23422
D_2O	2.00 E-09	19

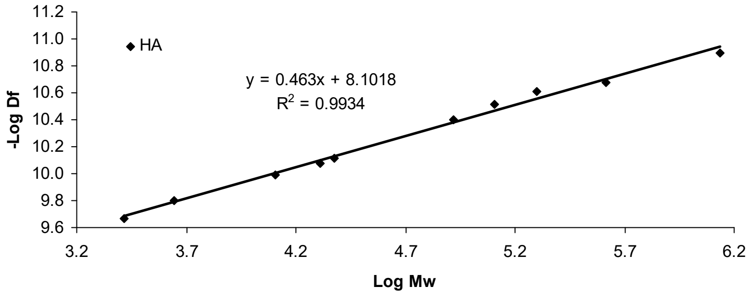

Figure S2: Decay line, D_f and Molecular Weight (MW) calculated from HA 100 mg L^{-1}. MW was calculated by interpolation using D_f from Cameron et al. 1972: Cameron, R. S., Thornton, B. K., Swift, R. S., Posner, A. M., Molecular weight and shape of humic acid from sedimentation and diffusion measurements on fractionated extracts. *European Journal of Soil Science* **197,** 23, 394-408.

Fluorescence spectroscopy

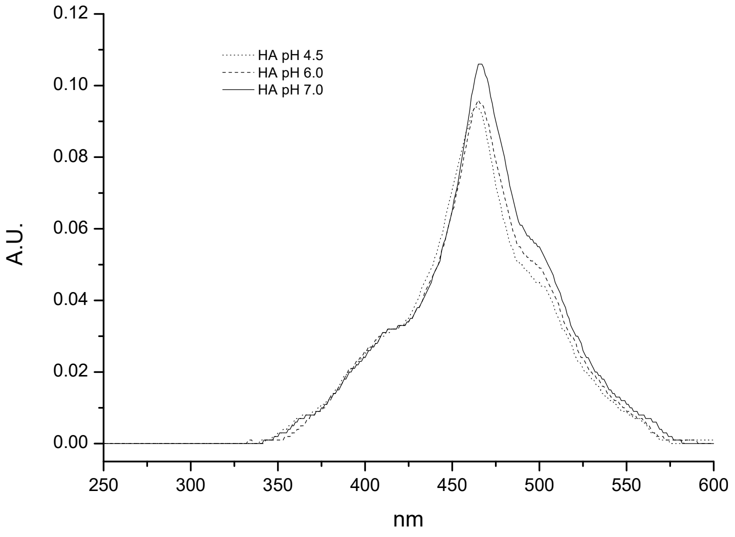

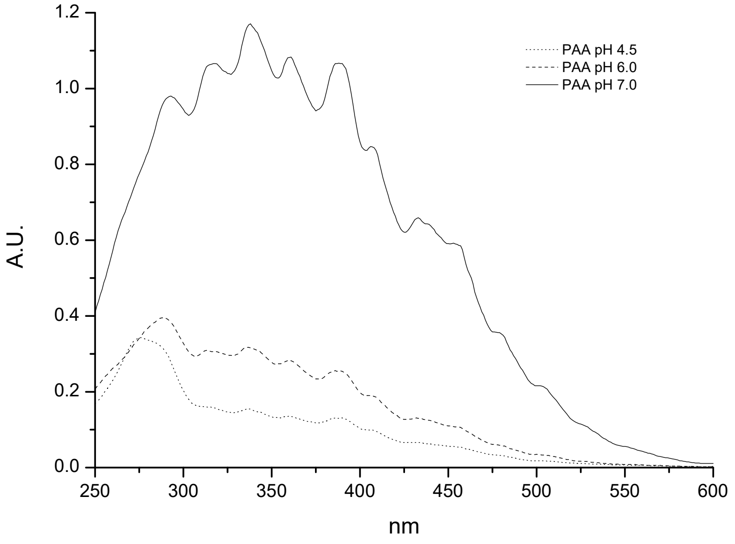

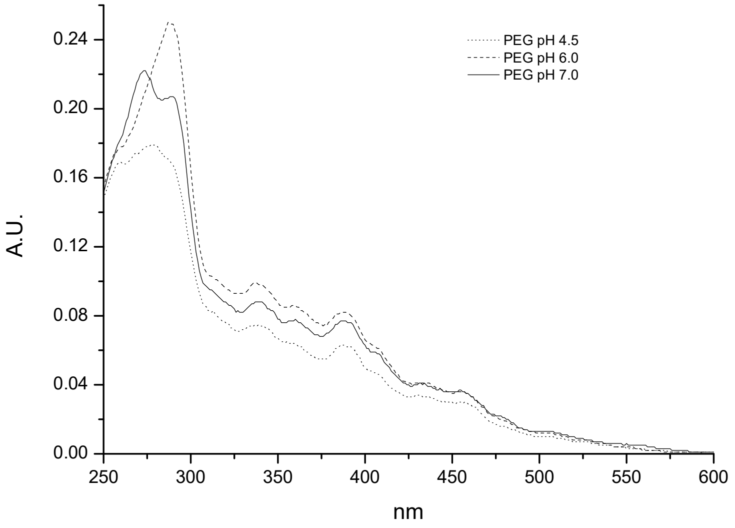

Figure S3. Synchronous fluorescence spectra for HA, PAA and PEG as a function of pH. Synchronous spectra were recorded between 250–600 nm wavelengths, with a 5-nm slit width on both monochromators. The scan speed of spectra 120 nm min^{-1} and resolution 0.5 nm.

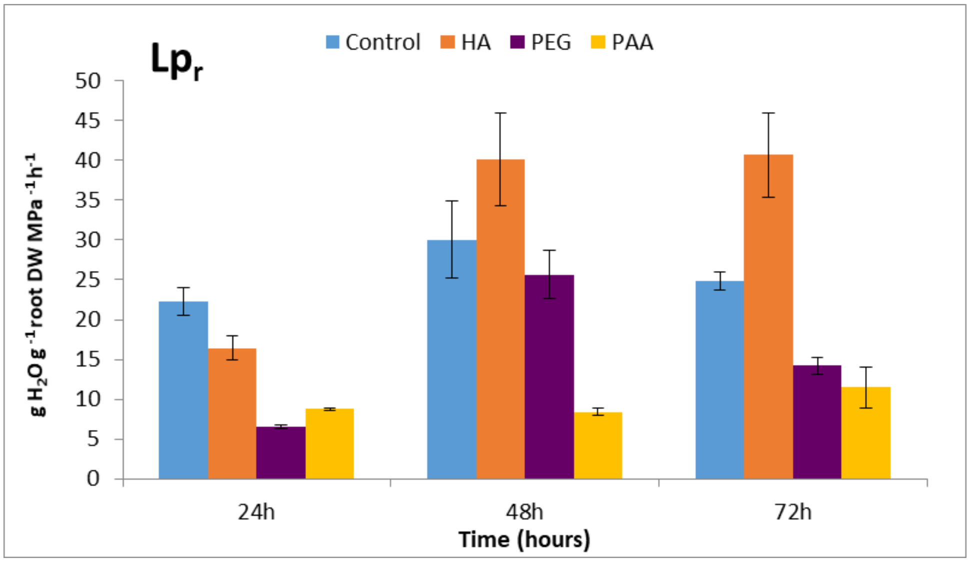

Figure S4: Lpr ± standard error (n=5). Time evolution of Lpr for the treated plants. Measurements were carried out after 24, 48 and 72 hours of treatment. Experiments were repeated three times.
